# Expression of annexin-A1 in blood and tissue leukocytes of leprosy patients

**DOI:** 10.1590/0037-8682-0277-2020

**Published:** 2020-11-25

**Authors:** Afonso Bezerra Ribeiro, Caroline Marques Caloi, Silvia Thais Sá Pimenta, Sudha Seshayyan, Srinivas Govindarajulu, Francisco José Dutra Souto, Amílcar Sabino Damazo

**Affiliations:** 1 Universidade Federal de Mato Grosso, Faculdade de Medicina, Programa de Pós-Graduação em Ciências da Saúde, Cuiabá, MT, Brasil.; 2 Universidade Federal de Mato Grosso, Faculdade de Medicina, Cuiabá, MT, Brasil.; 3 The Tamil Nadu Dr. MGR Medical University, Guindy, Chennai, India.; 4 The Tamil Nadu Dr. MGR Medical University, Department of Epidemiology, Guindy, Chennai, India.; 5 Universidade Federal de Mato Grosso, Faculdade de Medicina, Departamento de Clínica Médica, Cuiabá, MT, Brasil.; 6 Universidade Federal de Mato Grosso, Faculdade de Medicina, Departamento de Ciências Básicas em Saúde, Cuiabá, MT, Brazil.

**Keywords:** Leprosy, Annexin A1, Leukocytes, Tuberculosis, Lepromatous

## Abstract

**INTRODUCTION:**

In leprosy, immune system mediators that regulate the infectious process act in a complex manner and can lead to several clinical outcomes. To understand the behavior of these mediators we quantified the expression of annexin-A1 (ANXA1) in the peripheral blood and plasma as well as tissue leukocytes in all clinical forms of leprosy and compared with healthy controls.

**METHODS:**

Seventy healthy controls and 70 patients with leprosy, tuberculoid (TT) (n = 13), borderline tuberculoid (BT) (n = 15), borderline borderline (BB) (n = 13), borderline lepromatous (BL) (n = 15), and lepromatous leprosy (LL) (n = 14), were selected. Phenotyping of the lymphocyte cells and the intracellular expression of ANXA1 in leukocytes was performed by immunofluorescence. Plasma protein levels were determined by enzyme-linked immunosorbent assay.

**RESULTS:**

Histiocytes and CD4^+^ and CD8^+^ T cells in the skin of BL and LL patients had higher ANXA1 expression. ANXA1 expression was also high in circulating polymorphonuclear, monocytes, and CD4^+^ and CD8^+^ T cells in the blood of LL patients compared to those of TT, BT, BB, and BL patients, and these levels were similar to those in healthy controls. Plasma ANXA1 levels indicate an increase in paracrine release in patients with LL.

**CONCLUSIONS:**

The data indicate that ANXA1 expression is enhanced in the leukocytes and plasma of patients with LL, and may contribute to the inhibition of leukocyte action, leading to inadequate functioning of the immune system and thus contributing to the spread of *M. leprae* infection.

## INTRODUCTION

Leprosy is a chronic infectious disease caused by *Mycobacterium leprae*, an obligate intracellular bacterium that preferably infects macrophages and Schwann cells^1^ ^,^ ^2^. Leprosy is highly infectious but has low pathogenicity. However, a few of the infected individuals actually become ill, and thus leprosy is considered a disease of slow and insidious evolution, with multiple clinical outcomes that are dependent on the host immune response^3^. The clinical forms of leprosy, which were described by Ridley and Joplin^4^ based on clinical and histopathological criteria, are the following: tuberculoid (TT), borderline tuberculoid (BT), borderline borderline (BB), borderline lepromatous (BL), and lepromatous leprosy (LL)^4^ ^,^ ^5^.

The immune response to leprosy is complex and not fully understood. Molecular analyses can identify the presence of inflammatory regulatory molecules which could provide clarity regarding the host response mechanisms. An important molecule in this context is annexin-A1 (ANXA1), an anti-inflammatory protein which plays an important role in the regulation of leukocyte migration^6^, production of cytokines^7^, and activation of apoptosis^8^.

In the skin, ANXA1 is present in the epidermis, hair follicle, sebaceous gland, and vascular endothelium^9^. After an injury, ANXA1 expression is enhanced in the epidermis, neutrophils, monocytes, neovascular endothelial cells, and fibroblasts^9^. Other skin diseases, such as psoriasis vulgaris and keratosis follicularis, induce the expression of ANXA1^10^. ANXA1 is also upregulated in skin lesions of patients with leishmaniasis, especially in lymphocytes and macrophages^11^ ^,^ ^12^. Silva et al.^12^ showed that ANXA1 is highly expressed during exudative cellular reactions against *Leishmania* infection. In contrast, in the granulomatous reaction, the expression was attenuated, and parasite elimination was more favorable. 

Here, we quantified leukocyte populations, including neutrophils, monocytes, CD4^+^, CD8^+^, and regulatory T cells (TCD4^+^, TCD8^+^, and Tregs, respectively) in the peripheral blood of leprosy patients and compared to healthy control individuals. In addition, in order to assess the activation of immune system homoeostasis mechanisms, we quantified the expression of ANXA1 in the peripheral blood, plasma, and tissue leukocytes of leprosy patients.

## METHODS

### Study Participants

Patients of both sexes, ranging in age from 18 to 75 years, with a diagnosis of any clinical form of leprosy (n = 70) were eligible. Patients were recruited from the Infectious and Parasitic Diseases Clinic of Júlio Muller Teaching Hospital (HUJM), Cuiabá, Mato Grosso, Brazil, and were clinically categorized according to the criteria established by Ridley and Jopling as TT (n = 13), BT (n = 15), BB (n = 13), BL (n = 15), and LL (n = 14). 

The HC group was composed of healthy individuals without a history of leprosy and no known history of contact with leprosy (n = 70). 

### Exclusion criteria

We excluded individuals younger than 18 years and older 75 years, pregnant or lactating women, patients with concomitant infectious diseases or taking immunosuppressive agents, and patients with a diagnosis of leprosy who had previously started polychemotherapy.

### Ethical considerations

This study was approved by the Research Ethics Committee (CEP) of the University Hospital Júlio Muller (HUJM; protocol 733/CEP-HUJM/09) and met the requirements of Resolution 196/96 of the National Council of Health. The study was carried out in accordance with The Code of Ethics of the World Medical Association (Declaration of Helsinki). Informed consent was obtained from all patients.

### Blood and biopsy collection

A punch biopsy (4 mm) at the edge of the lesion was collected under anesthesia. Samples were immersed in 4% buffered paraformaldehyde (SIGMA, St. Louis, MO, USA) and processed in the Histology Laboratory of Faculty of Medicine, Universidade Federal of Mato Grosso (UFMT). Histological sections (3 μm) of the epithelial damaged regions were cut using a HIRAX M60 microtome (Carl Zeiss, Oberkochen, BW, Germany), arranged on histological slides, and stained with hematoxylin-eosin for the identification of epithelioid histiocytes, vacuolated or non-vacuolated histiocytes, multinucleated giant cells, lymphocytes, and plasmocytes. Fite-Faraco staining was also used to identify acid-fast bacilli (AFB). The bacillar index (BI) and histopathological alterations were used for classification of leprosy patients according to the criteria of Ridley and Jopling^4^.

Whole blood (5 mL) was collected from each patient by peripheral venipuncture in Vacutainer® tubes (Becton Dickson and Company, Franklin Lakes, NJ, USA) containing ethylenediamine tetra-acetic acid (EDTA) for blood smears. 

### Peripheral blood cell differential

The Neubauer hemocytometer chamber was used for total blood leukocyte count and the blood was diluted (1:10) in Turk's solution (SIGMA, St. Louis, MO, USA). Values are expressed as ×10^4^/mL leukocytes.

Differential counting of circulating leukocytes was performed on slides stained with the Fast Panoptic kit (AppliChem GmbH, Darmstadt, HE, Germany). One hundred leukocytes were counted for each sample by covering fields in a continuous zigzag manner. The smears were examined under a microscope with a 40× objective, identifying leukocytes in neutrophils, eosinophils, basophils, lymphocytes, and monocytes. 

### Enzyme-linked immunosorbent assay (ELISA)

A sample of 1 mL of whole blood was centrifuged to obtain blood plasma. ANXA1 levels in the diluted plasma (1:150) were determined by ELISA using a ready kit for assessing ANXA1 (USCN Life Sciences Inc., Houston, TX). The absorbance was measured using an SCA Absorbance Reader (Bio-Rad, Hercules, CA), and the reading of the samples was extrapolated according to a standard curve. Values are expressed as mean ± standard error of the mean (SEM) pg/mL plasma.

### Immunophenotypic evaluation by indirect immunofluorescence for neutrophils, monocytes/macrophages, TCD4+, TCD8+, and Tregs

Blood smear and skin section slides from leprosy patients were classified according to the clinical forms categorized by histopathological analysis as TT, BT, BB, BL, LL, and HC. 

For immunofluorescence, the samples were fixed in 4% paraformaldehyde buffered in phosphate buffered saline (PBS) (SIGMA, St. Louis, MO, USA) for 30 min at 4 °C and then incubated with the following reagents at 25°C, (a) washed three times with PBS for 5 min; (b) blocked with 3% hydrogen peroxide (H_2_O_2_) (SIGMA, St. Louis, MO, USA) diluted in methanol (SIGMA, St. Louis, MO, USA) and water for 1 h; (c) permeabilized by incubation with 0.4% Tween 20 (SIGMA, St. Louis, MO, USA) in PBS for 15 min; (d) blocked with 5% bovine serum albumin (BSA) (SIGMA, St. Louis, MO, USA) diluted in PBS for 1 h; (e) incubated for 18 h at 4 °C in a humid chamber with the following primary antibodies (diluted in 1% BSA): monoclonal mouse anti-myeloperoxidase (clone: 2C7, 1:100) (Invitrogen™, Carlsbad, CA, USA), polyclonal mouse anti-CD163 (clone EP152, 1:200) (Cell Marque, Rocklin, CA, USA), monoclonal mouse anti-human CD8 (clone: RPA-T8, 1:200) (eBioscience, San Diego, CA, USA), monoclonal mouse anti-human CD4 (clone: RPA-T4, 1:500), monoclonal sheep anti-CD25 (clone 9.14, 1:100) (Invitrogen™), and rat anti-FOXP3 (clone: FJK-16s, 1:200) (Invitrogen™); (f) washed with 1% BSA for 15 minutes; (g) incubated with the following secondary antibodies (diluted in 1% BSA) for 1 h at room temperature in a dark chamber: goat anti-mouse conjugated with Alexa Fluor 546® fluorochrome (1:50), goat anti-rat conjugated with Alexa Fluor 633® fluorochrome (1:50), and donkey anti-sheep conjugated with Alexa Fluor 350® fluorochrome (1:50) (Invitrogen™); 4’,6-diamidino-2-phenylindole (DAPI) (Invitrogen™, USA) was used as a nuclear marker; and finally, (h) slides were washed with PBS and mounted with PBS and glycerol (1:1) solution. 

The immune-labeled cells were identified with an AxioScopeA1 microscope (Carl Zeiss). For the quantification of neutrophils, monocytes, TCD4^+^, TCD8^+^, and Tregs, one hundred cells were counted in different fields for each individual, with the aid of an image analyzer, Software AxioVision (Carl Zeiss, Oberkochen, BW, Germany). 

### Evaluation of ANXA1 expression

The detection of endogenous ANXA1 on leukocytes was performed as previously described^13^. The slides were incubated with a rabbit anti-ANXA1 primary antibody (1:200 in 1% BSA) (Invitrogen™). A goat anti-rabbit antibody conjugated with Alexa Fluor 488® fluorochrome (Invitrogen, 1:50 in 1% BSA) was used as a secondary antibody. Immunolabeled cells were identified using an AxioScope A1 microscope (Carl Zeiss). ANXA1 was identified using the AxioVision Software (Carl Zeiss) and quantified by the mean optical density (MOD). The ANXA1 values for each cell were expressed as the mean ± SEM of each patient. In this way, it was possible to evaluate the mean leukocyte markings in each clinical type of leprosy. 

### Statistical analysis

Statistical comparison of the results was performed with the aid of GraphPad Prism software 5 (La Jolla, CA, USA) through one-way analysis of variance (ANOVA) with a Bonferroni post-test. The results are presented as mean ± SEM (standard error of the mean). A *p* value of less than 0.05 was considered statistically significant.

## RESULTS

### Leukocyte population profile in leprosy patients

**FIGURE 1:**
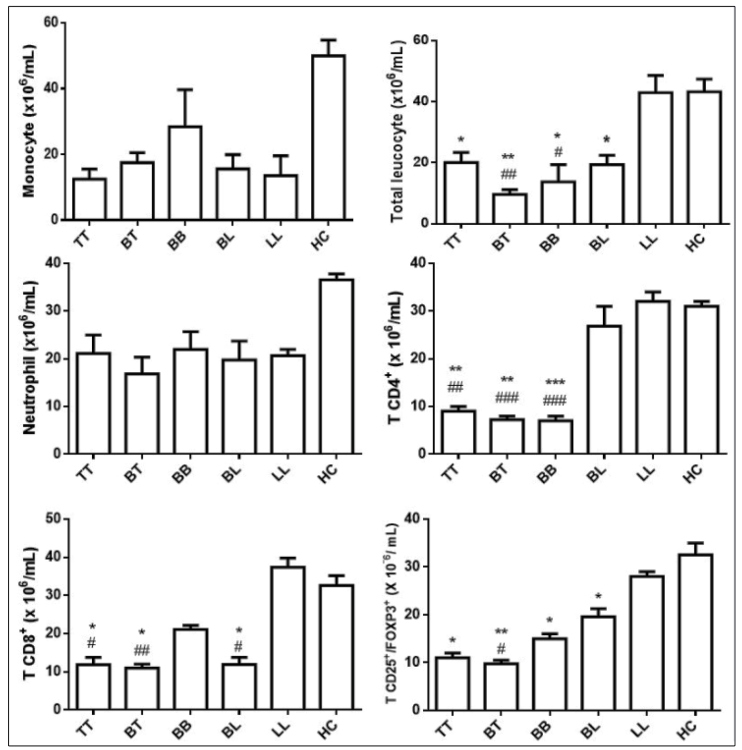
Analysis of total leukocytes, neutrophils, monocytes, and CD4^+^, CD8^+^, and regulatory T (CD25^+^/FOXP3^+^) cells in patients with the tuberculoid (TT), borderline tuberculoid (BT), borderline borderline (BB), borderline lepromatous (BL), and lepromatous leprosy (LL) clinical forms of leprosy and healthy controls (HC). The results are expressed as mean ± SEM and analyzed by one-way analysis of variance followed by a Bonferroni post-test, *p <0.05, **p <0.01, ***p <0.001 versus HC; #p <0.05, ##p <0.01, ###p <0.001 versus LL.

Analysis of total leukocytes (Figure 1) showed that TT (61.0%, *p* <0.05), BT (29.2%*, p* <0.01), BB (41.8%, *p* <0.05)*,* and BL (59.1%, *p* <0.05) patients presented reductions in the number of cells when compared to the HC group. On the other hand, LL patients presented no alterations in relation to HC.

Analysis of the patient leukocyte profile in all clinical forms showed that there was no statistical difference in the number of neutrophils and monocytes (Figure 1).

Differential analysis of lymphocytes indicated that there was a significant reduction in the number of TCD4^+^, TCD8^+^, and Tregs (CD4^+^: 33.4%, *p* <0.01 TT, 20.4%, *p* <0.01 for BT, 15%, *p* <0.05 for TT, 32.7%, *p* <0.05 for LB, and 35.8%, *p* <0.05 for BL; Tregs: 46.4%, *p* <0.05 for TT, 28.0% *p* <0.05 for BT, 45.0% *p* <0.05 for BB, and 53.5% *p* <0.05 for BL) in relation to the HC control, while LL patients did not present alterations (Figure 1).

Eosinophil and basophil counts were not considered in this analysis because their values were not altered.

### ANXA1 expression is up-regulated in different leukocytes of leprosy patients

**FIGURE 2:**
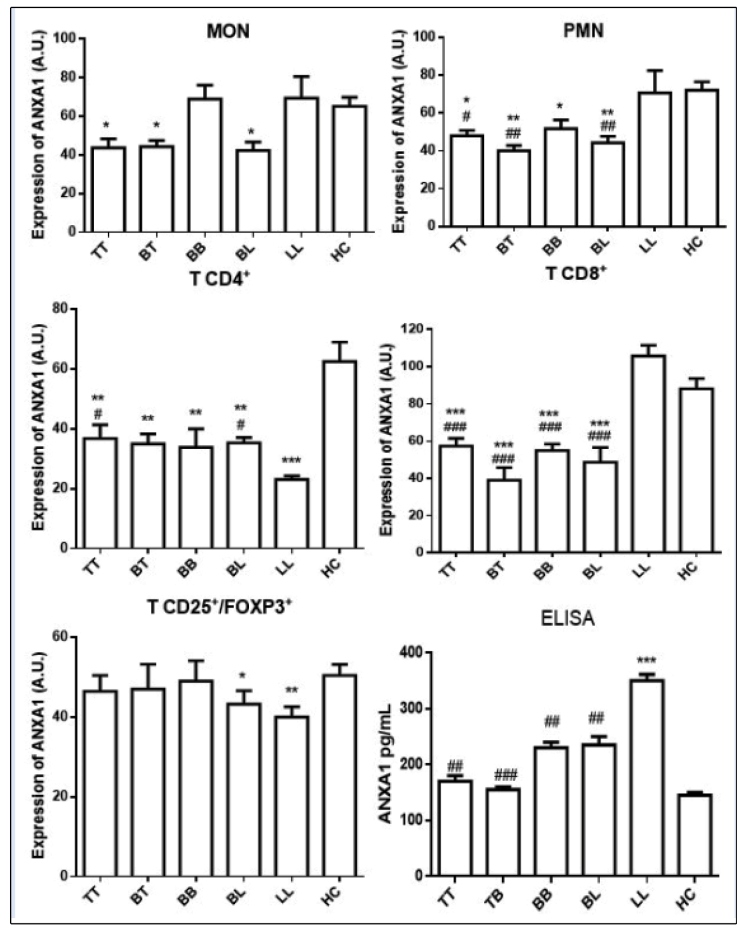
Intra-cytoplasmic expression of ANXA1 measured by indirect immunofluorescence and ELISA in leukocytes and the blood of patients with tuberculoid (TT), borderline tuberculoid (BT), borderline borderline (BB), borderline lepromatous (BL), and lepromatous leprosy (LL) leprosy and healthy controls (HC). Polymorphonuclear cells, monocytes, and CD4^+^, TCD8^+^, and regulatory (CD25^+^/FOXP3^+^) T cells were evaluated. The results are expressed as mean ± SEM (standard error of the mean) of arbitrary units (A.U.) and analyzed by one-way analysis of variance followed by a Bonferroni post-test, *p <0.05, **p <0.01, ***p <0.001 versus HC; #p <0.05, ##p <0.01, ###p <0.001 versus LL.

The expression of ANXA1 in polymorphonuclear cells was reduced in TT (65.3%, *p* <0.01), BT (55.4%, *p* <0.01), BB (71.6%, *p* <0.05)*,* and BL (*61.4%, p* <0.05) patients when compared to the HC group, which was not observed in LL patients (Figure 2). No statistically significant difference was observed in the analysis of monocytes (Figure 2).

The ANXA1 levels were reduced in TCD4^+^ in all clinical forms: TT (58.8%, *p* <0.01), BT (56.0%, *p* <0.01), BB (54.0%, *p* <0.05), BL (56.5%, *p* <0.001), and LL (*37.0%, p* <0.001) (Figure 2). However, in TCD8 cells, ANXA1 was reduced only in the TT (61.8%, *p* <0.001), BT (34.4%, *p* <0.001), BB (62.2%), and BL (*52.4%, p*<0.001) clinical forms. In contrast, LL patients had no altered expression of ANXA1 in these cells (Figure 2). 

In Treg cells, the expression of ANXA1 was significantly reduced in the BL (71.7%, *p*< 0.05) and LL (66.3%, *p*< 0.01) clinical forms. However, the same was not encountered in the TT, BT, and BB patients, as there were no statistically significant differences in the ANXA1 levels (Figure 2). 

Finally, the ANXA1 levels were evaluated in the leprosy patients, which indicated an increase in the paracrine release of this protein in the LL patients compared to the HC group (*p*<0.001) (Figure 2).

**FIGURE 3:**
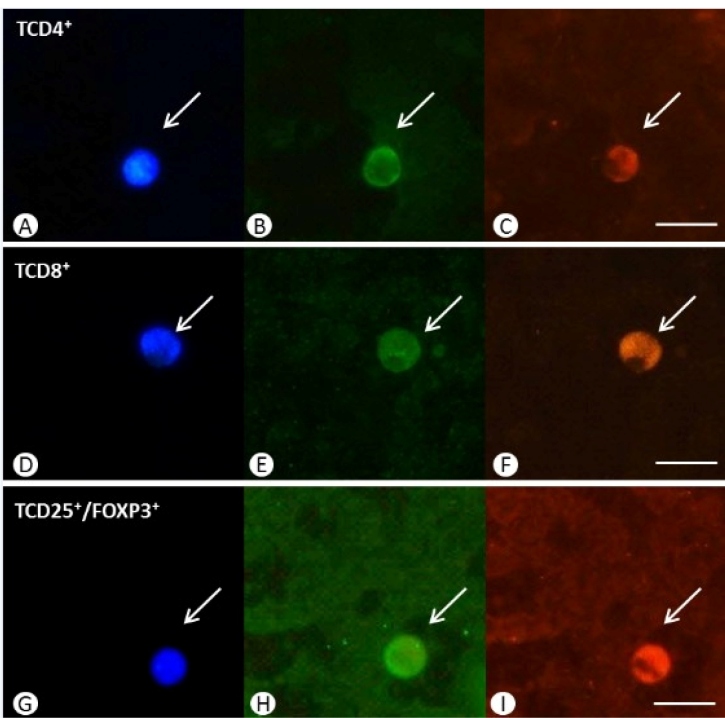
Molecular markers in peripheral blood smears of a patient with lepromatous leprosy. **(A, D, G)** Nucleolar labelling with DAPI. Immunofluorescent labeling for the: **(B, E, H)** Annexin A1 (ANXA1) protein; **(C, F)** CD8 and CD4 membrane markers; **(I)** CD25/ FOXP3 membrane marker. Scale bar = 100 μm.

Figure 3 illustrates the immunostaining pattern of the TCD4+, TCD8+, and Treg cells in LL patients.

### Annexin-A1 expression is up-regulated in different leukocytes in the skin lesions of leprosy patients

**FIGURE 4:**
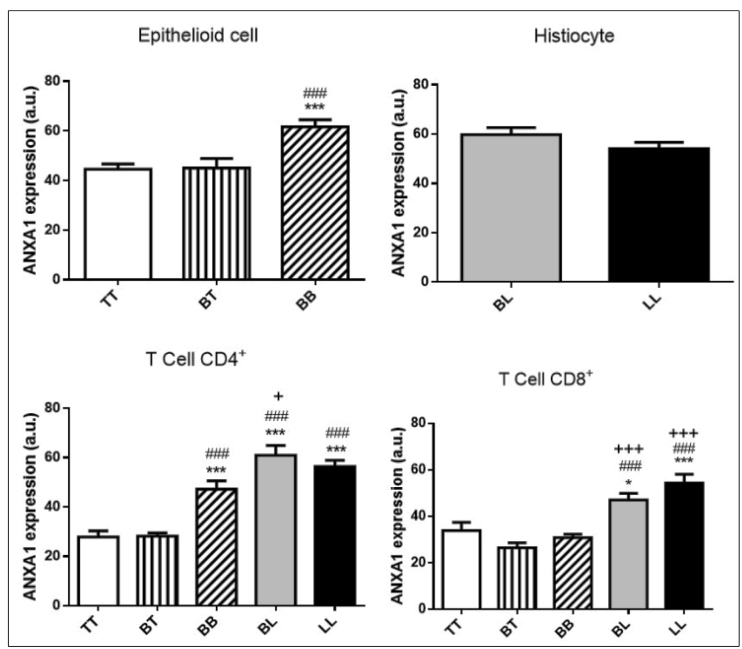
Analysis of intracellular expression of endogenous ANXA1 in cells present in the inflammatory site of leprosy patients. **(A)** Epithelioid cells. **(B)** Histiocytes. **(C)** CD4^+^ T cells. **(D)** CD8^+^ T cells. Data were analyzed by one-way analysis of variance and a Bonferroni post-test. a.u. = arbitrary units; *p <0.05. ***p <0.001 versus TT; ###p <0.001 versus BT; +p <0.05; +++p <0.001 versus BB.

In epithelioid cells, positive labeling of ANXA1 was observed in TT and BT patients, but in BB patients, the immunostaining was significantly higher (*p* <0.001) (Figure 4). Additionally, ANXA1 was expressed in histiocytes of patients with BL and LL, with no statistical differences between these clinical forms (Figure 4). In TCD4^+^ and TCD8^+^cells, ANXA1 expression was lower in BT and TT patients than in BB, BL, and LL patients (*p* <0.001) (Figure 4).

## DISCUSSION

Leprosy has several clinical outcomes due to scarring resulting from the uncontrolled inflammation, leading to neuronal damage and disability^1^ ^-^ ^3^ ^,^ ^5^. The work presented here indicates that endogenous ANXA1 acts as an inhibitory mediator for leukocytes targeting *M. leprae*. Thus, understanding the importance of endogenous anti-inflammatory ANXA1 in leukocyte regulation among leprosy patients can help prevent the worst outcomes in this disease. Our results suggest that endogenous ANXA1 contributes to suppressing inflammation not only at the site of the lesion but also appeared to have a more global effect through release into the blood. 

This study described the blood leukocyte profile in each clinical form of leprosy. The data showed an increasing number of TCD4^+^, TCD8^+^, and Treg cells in the blood and tissues of LL patients. These results provide a set of immunological biomarkers for the clinical spectrum of leprosy. Other studies have reported similar results^14^ ^-^ ^17^. The infection caused by *M. leprae* induces a specific immune response that is directly associated with the clinical form existing in the individual^10^ ^-^ ^15^. Leukocyte recruitment to an inflammatory site is mainly triggered by a strong Th1 response, resulting in granuloma formation, predominantly formed by histiocytes and lymphocytes, in an attempt to eliminate the bacillus^21^ ^-^ ^28^. However, some patients fail to fight against the bacillus. In particular, Tregs might be responsible for T cell downregulation, leading to the antigen-specific anergy associated with LL patients^14^.

This is the first study describing the expression of ANXA1 in peripheral blood and skin tissue leukocytes of patients with leprosy. ANXA1 is an important protein modulator of acute inflammatory activity involved in the inhibition of leukocyte extravasation to the site of inflammation^6^ ^,^ ^7^ ^,^ ^29^. This molecule has been studied to elucidate the mechanisms that operate in the host during the resolution of the inflammatory process^6^ ^,^ ^7^ ^,^ ^13^. In this study, tuberculoid patients had reduced expression of ANXA1 in circulating polymorphonuclear cells when compared to lepromatous patients. Neutrophils, eosinophils, monocytes, lymphocytes, and epithelial cells physiologically express ANXA1^30^. The expression of this protein is dependent on inflammatory stimuli. Tuberculoid patients have a low level of bacilli. Therefore, blood polymorphonuclear cells express less ANXA1 in these patients. Silva et al.^11^ observed similar results, showing that ANXA1 is highly expressed in uncontrolled leishmaniasis lesions. However, in a lesion with granuloma formation, the expression was reduced. 

In circulating monocytes, ANXA1 expression was similar in all clinical forms of leprosy. However, skin histiocytes expressed more ANXA1 in patients with LL. Several studies describe ANXA1 as a critical protein in the macrophage phagocytic process^31^. According to Scanell et al.^32^ and Tzelepis et al.^29^, apoptotic neutrophils release ANXA1, activating macrophages to perform phagocytosis. The absence of ANXA1 in transgenic animals reduces the phagocytic capacity of macrophages^33^. In an experimental study using a granulomatous inflammation model in mice, Gibbs et al.^34^ found that macrophages present a transient expression of ANXA1, with a peak at 7 and 28 days and a lower expression at 14 days after experimental procedure. 

In this study, the ANXA1 levels were also increased in TCD4^+^, TCD8^+^, and Treg cells from the blood and skin tissues of BL and LL patients, when compared to TT, BT, and BB patients. These data might indicate a role of ANXA1 in the activation of lymphocytes in the different clinical forms of leprosy, which may influence the effectiveness of the response to the bacilli. LL patients develop a Th2 immune response and are unable to contain the spread of the bacillus^16^ ^-^ ^22^. ANXA1 regulates TCD4^+^ and TCD8^+^ cell migration to inflammatory sites^35^ ^-^ ^36^. Another study using transgenic animals deficient in ANXA1 observed that TCD4^+^ and TCD8^+^ cells become more active in the lungs of mice infected with *Mycobacterium tuberculosis*, leading to the destruction of the bacillus^29^. Moreover, activated TCD8^+^ cells express lower levels of ANXA1^7^ ^,^ ^37^. Thus, increased levels of ANXA1 in TCD8^+^ cells in LL patients could inhibit the cytotoxic action of this cell and may lead to ineffective elimination/containment of the bacillus. A study using transgenic mice indicated that T cell-expressed ANXA1 functions to attenuate T cell-driven inflammatory responses via T cell-intrinsic effects on intracellular signaling, proliferation, and Th1/Th17 cytokine release^36^. Only a few studies have described ANXA1 expression in Treg cells^38^ ^,^ ^39^. These data indicated that ANXA1 could enhance the inhibitory function of Treg cells. We believe that the high ANXA1 expression in Treg cells might be associated with the anergy associated with LL patients.

In conclusion, ANXA1 protein was characterized as a possible regulator of the infectious process induced by *M. leprae*, indicating that higher expression of this protein in leukocytes and in the plasma might down-regulate the pro-inflammatory potential in BB, BL, and LL patients, leading to the proliferation of *M. leprae*. In TT and BT patients, ANXA1 had lower levels, which could contribute to leukocyte activation, favoring the elimination of the bacilli.
